# Flt1 produced by lung endothelial cells impairs ATII cell transdifferentiation and repair in pulmonary fibrosis

**DOI:** 10.1038/s41419-023-05962-2

**Published:** 2023-07-15

**Authors:** Maria Concetta Volpe, Giulio Ciucci, Giulia Zandomenego, Roman Vuerich, Nadja Anneliese Ruth Ring, Simone Vodret, Francesco Salton, Pietro Marchesan, Luca Braga, Thomas Marcuzzo, Rossana Bussani, Andrea Colliva, Silvano Piazza, Marco Confalonieri, Serena Zacchigna

**Affiliations:** 1 Cardiovascular Biology Laboratory, International Centre for Genetic Engineering and Biotechnology (ICGEB), Trieste, Italy; 2 Department of Life Sciences, University of Trieste, Trieste, Italy; 3 Functional Cell Biology Laboratory, International Centre for Genetic Engineering and Biotechnology (ICGEB), Trieste, Italy; 4 Pulmonology Unit, University Hospital of Cattinara, Trieste, Italy; 5 Department of Medicine, Surgery and Health Sciences, University of Trieste, Trieste, Italy; 6 Computational Biology Laboratory, International Centre for Genetic Engineering and Biotechnology (ICGEB), Trieste, Italy; 7 Present Address: Ludwig Boltzmann Gesellschaft Research Group Senescence and Healing of Wounds, Vienna, Austria

**Keywords:** Respiratory tract diseases, Mechanisms of disease, Adult stem cells, Regenerative medicine

## Abstract

Pulmonary fibrosis is a devastating disease, in which fibrotic tissue progressively replaces lung alveolar structure, resulting in chronic respiratory failure. Alveolar type II cells act as epithelial stem cells, being able to transdifferentiate into alveolar type I cells, which mediate gas exchange, thus contributing to lung homeostasis and repair after damage. Impaired epithelial transdifferentiation is emerging as a major pathogenetic mechanism driving both onset and progression of fibrosis in the lung. Here, we show that lung endothelial cells secrete angiocrine factors that regulate alveolar cell differentiation. Specifically, we build on our previous data on the anti-fibrotic microRNA-200c and identify the Vascular Endothelial Growth Factor receptor 1, also named *Flt1*, as its main functional target in endothelial cells. Endothelial-specific knockout of *Flt1* reproduces the anti-fibrotic effect of microRNA-200c against pulmonary fibrosis and results in the secretion of a pool of soluble factors and matrix components able to promote epithelial transdifferentiation in a paracrine manner. Collectively, these data indicate the existence of a complex endothelial-epithelial paracrine crosstalk in vitro and in vivo and position lung endothelial cells as a relevant therapeutic target in the fight against pulmonary fibrosis.

## Introduction

Pulmonary fibrosis is a progressive disease in which scarring of normal lung tissue leads to death from respiratory failure [1]. The two currently available drugs, pirfenidone and nintedanib, have only modest effect on survival [2]. The lack of effective therapies is likely the consequence of a poor understanding of the pathogenetic mechanisms that trigger disease onset and progression, particularly in its idiopathic form (Idiopathic Pulmonary Fibrosis, IPF) [3].

While lung fibroblasts are clearly the ultimate actors in the deposition of fibrotic tissue [4, 5], emerging evidence indicates that IPF should be considered an epithelial-driven disease [6]. The alveolar epithelium is composed of fully differentiated alveolar type I (ATI) cells, which perform gas exchange, and alveolar type II cells (ATII), which produce surfactant proteins and act as progenitor cells, giving rise to new ATI cells upon injury. The loss of regenerative potential of ATII cells could represent a major driver of IPF development [7]. Recent evidence indicates that alveologenesis and ATII cell activation depend on their close physical and functional interaction with lung endothelial cells (ECs) through the secretion of angiocrine signals [8, 9], although to our knowledge a role of the lung endothelium in IPF has never been investigated.

Among the regulators of alveolar epithelial function are microRNAs (miRNAs), approximately 22 nucleotide long non-coding RNA molecules, which function through the repression of multiple target messenger RNAs (mRNAs) [10]. Several miRNAs have been implicated in lung epithelial repair, epithelial-mesenchymal transition (EMT), and collagen production, all of which are relevant pathological mechanisms in IPF [11, 12]. Microarray analysis of lung samples, from IPF patients and healthy individuals, have confirmed a divergent miRNA profile between patients and controls [13]. We recently demonstrated that ATII cells isolated from IPF patients fail to transdifferentiate into ATI cells [14] and undergo both EMT and senescence. Delivery of miR-200c-3p to ATII cells from IPF patients rescued their transdifferentiation capacity, supporting the therapeutic potential of miR-200c in IPF [14, 15]. Whether miR-200c can also act on other cell types that reside in the lung remains elusive.

Here, we induce pulmonary fibrosis in mice using bleomycin and show that ATII cells from these mice do not efficiently transdifferentiate into ATI cells. We also describe a novel mechanism by which miR-200c-3p protects from and reverts pulmonary fibrosis, identifying the endothelial Flt1 receptor as a relevant target, and demonstrating a complex endothelial-to-epithelial paracrine crosstalk.

## Material and methods

Additional details are provided as supplementary information.

### Primary cells and miRNA transfection

ATII cells were isolated from distal lungs of C57BL/6 mice as reported [16]. Primary murine and human ECs were isolated from adult lung of C57BL/6 mice or human IPF patients and healthy donors, as described [14, 17]. EC transfection was performed in 96-well plates using Lipofectamine RNAiMAX (Thermo Fisher Scientific).

### Animal experiments

C57BL/6 and Cdh5-Cre; *Flt1 fl/fl* mice were injected intratracheally with bleomycin (1 U/kg), while miR-200c-3p was administered at 6 μg/kg.

### Histology and microscopy

Cells were fixed with 4% PFA and incubated with primary and secondary antibodies. Images were acquired using Operetta high content screening microscope (Perkin Elmer). For fibrosis quantification, tissues were stained using Masson-Trichrome (Bio Optica).

### qRT-PCR

RNA was extracted using Trizol (Invitrogen) and cDNA was prepared using the First strand cDNA synthesis kit (Thermo Scientific) as described [18].

### Mass spectrometry

Protein samples (20 μg) were digested with Lys-C (Promega, #VA1170), desalted and lyophilized for LC-MS/MS analysis on an Easy-nLC 1200 system (Thermo Fisher Scientific).

### Statistical analysis

Two-way ANOVA statistics followed by Dunnet’s multiple comparison test was used for multiple datasets whereas one-way ANOVA and unpaired Student’s *t*-test were used to compare three or two groups of data, respectively. Significant values are indicated by asterisks, where *p < 0.05, **p < 0,01, ***p < 0,001, ****p < 0,0001.

## Results

### Bleomycin treatment in mice recapitulates the transdifferentiation defect observed in ATII cells harvested from IPF patients

**Fig. 1:**
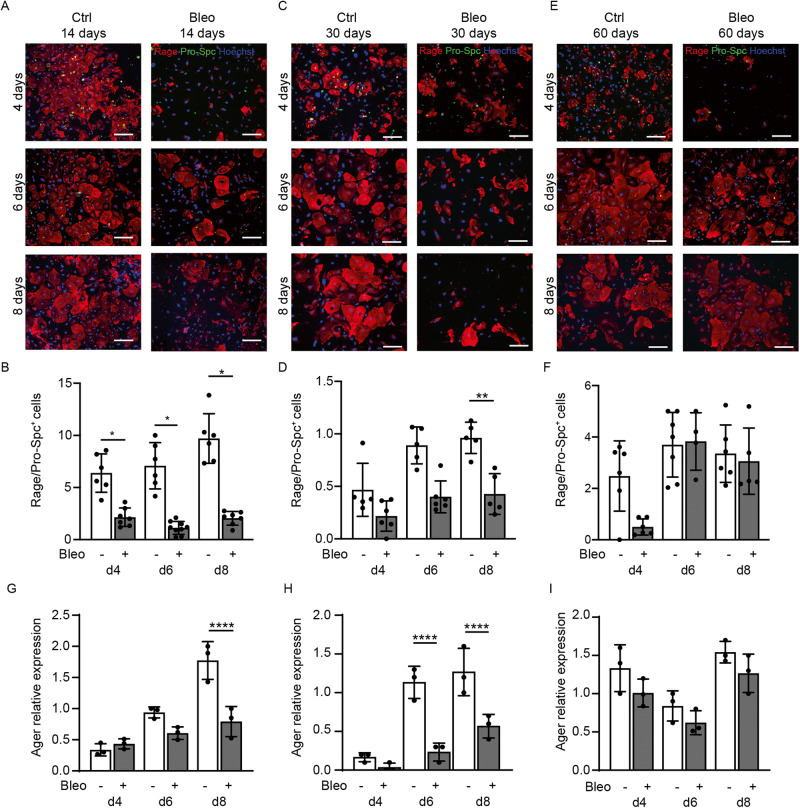
ATII cells from bleomycin-treated mice show impaired transdifferentiation. **A** Representative immunofluorescence images of primary murine ATII cells harvested at 14 days after PBS or bleomycin administration and kept in culture for the indicated time points. ATII and ATI cells were stained for Pro-Spc (green) and Rage (red), respectively. Nuclei were counterstained with Hoechst (blue). **B** Quantification of the transdifferentiation of ATII into ATI cells, shown as the ratio between Rage^+^ at the indicated time point and Pro-Spc^+^ cells at day 2. **C** Representative immunofluorescence images of primary murine ATII cells harvested at 30 days after PBS or bleomycin administration and kept in culture for the indicated time points. ATII and ATI cells were stained for Pro-Spc (green) and Rage (red), respectively. Nuclei were counterstained with Hoechst (blue). **D** Quantification of the transdifferentiation of ATII into ATI cells, shown as the ratio between Rage^+^ at the indicated time point and Pro-Spc^+^ cells at day 2. **E** Representative immunofluorescence images of primary murine ATII cells harvested at 60 days after PBS or bleomycin administration and kept in culture for the indicated time points. ATII and ATI cells were stained for Pro-Spc (green) and Rage (red), respectively. Nuclei were counterstained with Hoechst (blue). **F** Quantification of the transdifferentiation of ATII into ATI cells, shown as the ratio between Rage^+^ at the indicated time point and Pro-Spc^+^ cells at day 2. **G**–**I** Real-time PCR quantification of the expression levels of the ATI cell marker *Ager* (gene coding for Rage) by ATII cells harvested at 14 (**G**), 30 (**H**) and 60 (**I**) days after PBS or bleomycin administration and kept in culture for the indicated time points. Data are normalized on *Gapdh*. Scale bar in **A**, **C**, **E**, 100 μm. Data in **B**, **D**, **F**, **G**, **H**, and **I** are shown as mean ± S.E.M (*n* ≥ 3). Statistical significance was determined using two-way ANOVA, *P < 0.05, **P < 0.01, ****P < 0.0001.

Pulmonary fibrosis in mice is commonly modeled by intra-tracheal administration of bleomycin [4, 5]. First, we assessed whether this treatment reproduces the ATII transdifferentiation impairment observed in human IPF cells [14]. We isolated ATII cells from mouse lungs at 14, 30, and 60 days after bleomycin administration. Cell purity at different steps of the isolation procedure was assessed by both Western Blot and immunofluorescence analysis (Supplementary Fig. 1), showing >70% of ATII cells in the final cell preparation. We then evaluated their transdifferentiation into ATI cells by staining them for Pro-Surfactant protein C (Pro-Spc) and receptor of advanced glycation end-product (Rage), which label ATII and ATI cells, respectively. As expected, control ATII cells efficiently transdifferentiated into ATI cells. When kept in culture for 8 days, they progressively lost the ATII marker Pro-Spc, and upregulated the ATI marker Rage, becoming large and flattened. In contrast, ATII cells from lungs exposed to bleomycin for either 14 or 30 days, showed a drastic impairment in transdifferentiation, resulting in a 3-fold decrease in the number of fully transdifferentiated ATI cells at day 8 (Fig. 1A–D). This transdifferentiation capacity was rescued when ATII cells were harvested after 60 days after in vivo bleomycin administration (Fig. 1E, F), in line with the almost complete recovery of lung structure observed by histological analysis (Supplementary Fig. 2). Consistent results were obtained by quantifying the expression levels of ATII (*Sftpa1* and *Sftpc*) and ATI (*Podoplanin* and *Ager*) markers by RT-PCR (Fig. 1G–I and Supplementary Fig. 3).

### miR-200c-3p prevents and reverts bleomycin-induced pulmonary fibrosis

**Fig. 2:**
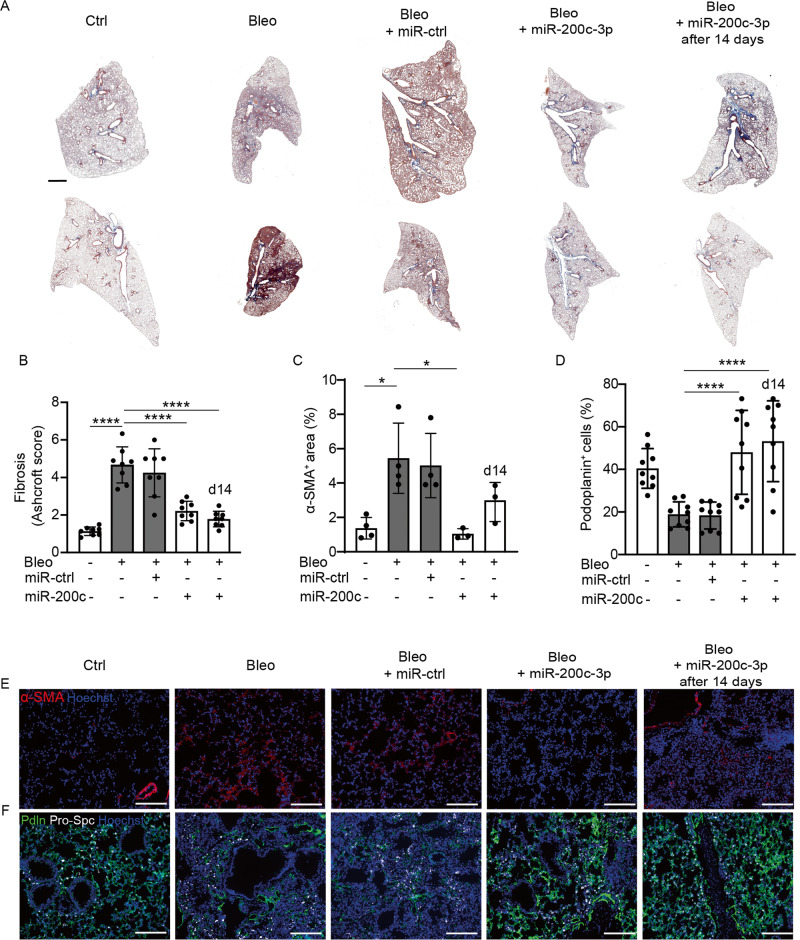
In vivo delivery of miR-200c-3p inhibits fibrosis and preserves the number of ATI cells in the lung. **A** Representative images of whole lung sections stained with Masson-Trichrome to visualize collagen fibers in blue at 30 days after the administration of the indicated treatments. **B** Quantification of pulmonary fibrosis using the Ashcroft score. **C** Quantification of the area covered by α-SMA^+^ activated fibroblasts. **D** Quantification of the percentage of Podoplanin^+^ cells. **E** Representative immunofluorescence images of lung sections stained for α-SMA. Nuclei were counterstained with Hoechst (blue). **F** Representative immunofluorescence images of lung sections stained for Pro-Spc (white) and Podoplanin (Pdln, green) to label ATII and ATI cells, respectively. Nuclei were counterstained with Hoechst (blue). Scale bar in **A**, 1 mm; in **E**, **F**, 100 μm. Data in **B**–**D** are shown as mean ± S.E.M (*n* ≥ 3). Statistical significance was determined using one-way ANOVA, *P < 0.05, ****P < 0.0001.

Having confirmed the suitability of the bleomycin model of pulmonary fibrosis in reproducing the transdifferentiation defect of alveolar epithelial cells, we aimed at assessing the therapeutic potential of miR-200c-3p, which we have previously shown to rescue the phenotype of human ATII cells from IPF patients through modulation of EMT [14]. First, we optimized in vivo delivery of miRNAs to murine lungs by aerosol, using a fluorescent miRNA (Supplementary Fig. 4A, B) and selected 6 μg/Kg as the minimal dose ensuring the highest efficiency of delivery (about 30% of transfected cells). Then, we delivered miR-200c-3p, which resulted in its 20-fold up-regulation at day 3 (Supplementary Fig. 4C). Having proven efficient miRNA delivery by aerosol, we administered either miR-200c-3p or a control miRNA immediately after bleomycin and assessed the development of pulmonary fibrosis by Masson trichrome staining. As shown in Fig. 2A, bleomycin administration resulted in marked inflammatory infiltration and collagen deposition at 1 month. The control miRNA had no effect on the extent of pulmonary fibrosis. In contrast, mice that received miR-200c-3p developed milder disease, with attenuated collagen deposition. Quantification of lung damage by the Ashcroft score showed a 3-fold reduction in the level of fibrosis in miR-200c-treated animals compared to controls (Fig. 2B). Consistent data were obtained by using anti-α-smooth muscle actin (α-SMA) antibodies to label activated fibroblasts, the main source of collagen, and quantifying their relative abundance in the various groups (Fig. 2C, E).

Next, we aimed at assessing whether miR-200c-3p was able to revert established pulmonary fibrosis. Thus, we delivered miR-200c-3p 14 days after bleomycin administration and sacrificed animals after an additional 16 days. Also in this case, miR-200c-3p significantly reduced pulmonary fibrosis and fibroblast activation, resulting in preservation of alveolar structure (right panel in Fig. 2A, E and quantification in Fig. 2B, C). We stained the same lungs for the ATII marker Pro-Spc and for Podoplanin, which labels ATI cells. As shown in Fig. 2D, F bleomycin injection resulted in a significant loss of ATI cells, consistent with the disruption of the alveolar structure reported by the Ashcroft score. In contrast, delivery of miR-200c-3p rescued the number of Podoplanin^+^ ATI cells, when administered in both preventive and therapeutic approaches (Fig. 2D, F).

Thus, aerosol delivery of miR-200c-3p both prevents and reverts pulmonary fibrosis induced by bleomycin. The therapeutic effect of miR-200c-3p on established pulmonary fibrosis suggests that its mechanism of action extends beyond inhibition of EMT, which mainly happens during the onset of pulmonary fibrosis.

### miR-200c-3p delivery to lung endothelial cells promotes ATII transdifferentiation through downregulation of *Flt1*

**Fig. 3:**
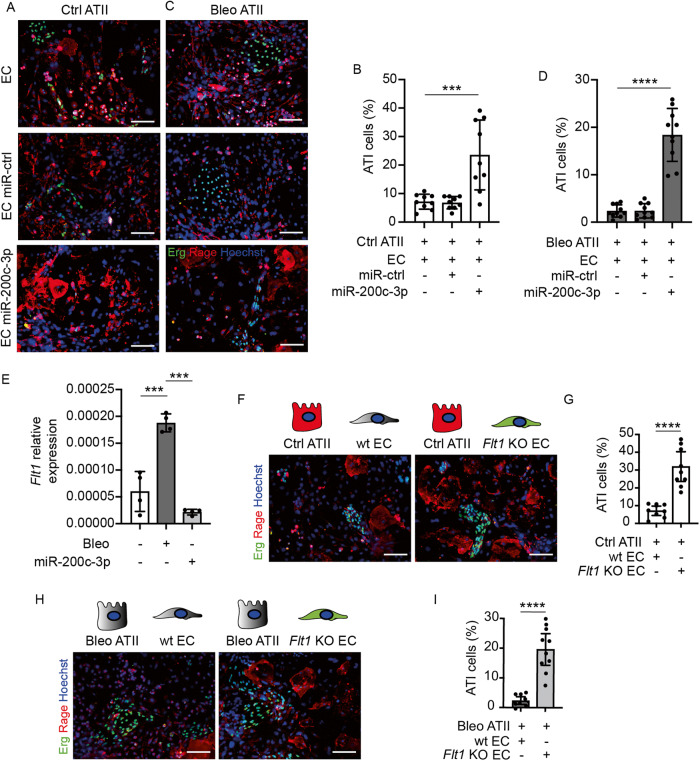
miR-200c-3p delivery to lung endothelial cells promotes ATII transdifferentiation through down-regulation of *Flt1*. **A** Representative immunofluorescence images of control ATII cells (stained for Rage, red) co-cultured with ECs (stained for Erg, green) either untreated or transfected with the indicated miRNAs. **B** Quantification of fully differentiated (>1500 µm^2^) Rage^+^ ATI cells, expressed as a percentage of Rage^+^ cells, in the indicated conditions; *n* ≥ 9. **C** Representative immunofluorescence images of ATII cells from bleomycin-treated mice (stained for Rage, red) co-cultured with ECs (stained for Erg, green) either untreated or transfected with the indicated miRNAs; *n* ≥ 9. **D** Quantification of fully differentiated (>1500 µm^2^) Rage^+^ ATI cells, expressed as a percentage of Rage^+^ cells, in the indicated conditions; *n* ≥ 9. **E** Real-time PCR quantification of the expression levels of *Flt1* in lungs harvested from control mice and mice treated with bleomycin, either in the presence or in the absence of miR-200c. Results are normalized to *Gapdh* expression; *n* = 4. **F** Representative immunofluorescence images of control ATII cells kept in culture with either wt or *Flt1* KO EC, stained for Erg (green) and Rage (red), respectively. Nuclei were counterstained with Hoechst (blue). **G** Quantification of fully differentiated (>1500 µm^2^) Rage^+^ ATI cells, expressed as a percentage of Rage^+^ cells, in the indicated conditions; *n* = 9. **H** Representative immunofluorescence images of ATII cells from bleomycin-treated mice, kept in culture with either wt or *Flt1* KO EC, stained for Erg (green) and Rage (red), respectively. Nuclei were counterstained with Hoechst (blue). **I** Quantification of fully differentiated (>1500 µm^2^) Rage^+^ cells ATI cells from bleomycin mice, expressed as a percentage of Rage^+^ cells, in the indicated conditions; *n* ≥ 9. Scale bar in **A**, **C**, **F**, **H**, 100 μm. Data in **B**, **D**, **E**, **G**, and **I** are shown as mean ± S.E.M. Statistical significance was determined using one-way ANOVA, ***P < 0.001, ****P < 0.0001.

To investigate additional mechanisms by which miR-200c-3p could prevent pulmonary fibrosis, we interrogated the miRNA target prediction software miRTarBase to identify its direct targets in murine cells. We found three targets that are experimentally validated by luciferase 3’UTR reporter assay: *Flt1*, *Zeb1* and *Zeb2* [19, 20]. As these genes, and in particular *Flt1*, are abundantly expressed by endothelial cells (ECs), we hypothesized that these cells might be involved in the therapeutic effect exerted by miR-200c-3p. Thus, we established a co-culture system, in which we transfected either miR-200c-3p or a control miRNA into primary lung ECs and then added ATII cells to the same plates. At 4 days, we observed that ECs treated with miR-200c-3p significantly promoted ATII cell transdifferentiation, resulting in an increased number of fully differentiated (>1500 µm^2^) Rage^+^ cells, compared to both control miRNA and untreated ECs (Fig. 3A, B). This effect was even more evident when we repeated the experiment with ATII cells harvested from bleomycin-treated mice. As shown in Fig. 3C, D, delivery of miR-200c-3p to ECs resulted in increased epithelial transdifferentiation.

### *Flt1* depletion in ECs enhances ATII transdifferentiation through the release of angiocrine factors

To identify the main targets of miR-200c-3p in ECs, we isolated lung ECs and transfected them with miR-200c-3p, a control miRNA and siUBC (to check and normalize for transfection efficiency). We performed qPCR analysis and found that *Zeb1*, *Zeb2* and, most evidently, *Flt1* were significantly down-regulated in miR-200c-transfected cells (Supplementary Fig. 5).

Among these targets, we focused on *Flt1*, as it is abundantly and almost exclusively expressed by ECs and it is highly up-regulated in both mouse and human fibrotic lungs [21]. Moreover,

*Flt1* showed the highest suppression by miR-200c-3p both in cultured ECs and in murine lungs (Fig. 3E and Supplementary Fig. 5).

To evaluate the role of *Flt1* in the crosstalk between alveolar endothelial and epithelial cells, we crossed Cdh5-ERT2 with Flt1^*flox/flox*^ mice to selectively knockout *Flt1* in ECs upon Tamoxifen administration (*Flt1 KO*^*iEC*^). Then, we isolated ECs from either wild type (wt) or *Flt1 KO*^*iEC*^ mice and cultured them together with ATII cells. As shown in Fig. 3F, G, *Flt1 KO* ECs promoted the transdifferentiation of ATII cells at an efficiency even higher than miR-200c-3p. As before, the effect was even more pronounced when ATII cells were harvested from fibrotic lungs (Fig. 3H, I).

**Fig. 4:**
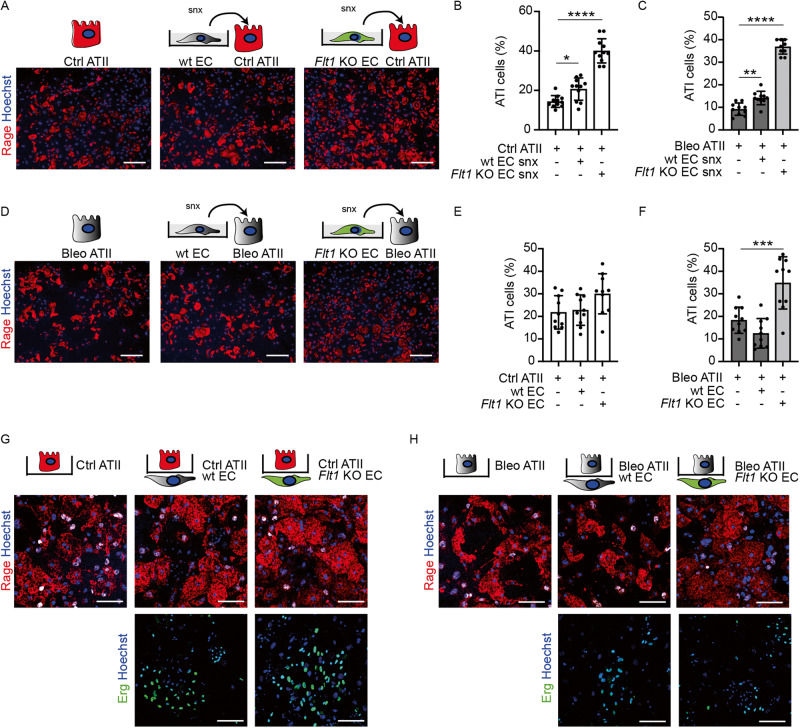
Endothelial Flt1 depletion in endothelial cells improves ATII transdifferentiation. **A** Representative immunofluorescence images of control ATII cells conditioned with wt or *Flt1* KO ECs supernatants, stained for Rage (red). Nuclei were counterstained with Hoechst (blue). **B** Quantification of fully differentiated (>1500 µm^2^) Rage^+^ ATI cells, expressed as a percentage of Rage^+^ cells, in the indicated conditions; *n* = 11. **C**. Quantification of fully differentiated (>1500 µm^2^) Rage^+^ ATI cells from bleomycin-treated mice, expressed as a percentage of Rage^+^ cells, in the indicated conditions; *n* = 10. **D** Representative immunofluorescence images of ATII cells from bleomycin-treated mice, conditioned with wt or Flt1 KO ECs supernatants, stained for Rage (red). Nuclei were counterstained with Hoechst (blue). **E** Quantification of fully differentiated (>1500 µm^2^) Rage^+^ ATI cells, expressed as a percentage of Rage^+^ cells, in the indicated conditions; *n* = 10. **F** Quantification of fully differentiated (>1500 µm^2^) Rage^+^ ATI cells from bleomycin-treated mice, expressed as a percentage of Rage^+^ cells, in the indicated conditions; *n* = 10. **G** Representative immunofluorescence images of control ATII cells in contact with wt or *Flt1* KO ECs cells, stained for Rage (red) and Erg (green), respectively. Nuclei were counterstained with Hoechst (blue). **H** Representative immunofluorescence images of ATII cells from bleomycin-treated mice in contact with wt or *Flt1* KO ECs cells, stained for Rage (red) and Erg (green), respectively. Nuclei were counterstained with Hoechst (blue). Scale bar in **A**, **D**, **G**, **H**, 100 μm. Data in **B**, **C**, **E**, and **F** are shown as mean ± S.E.M (*n* = 10). Statistical significance was determined using one-way ANOVA, *P < 0.05, **P < 0.01, ***P < 0.001, ****P < 0.0001.

These data show that *Flt1* KO in ECs promotes transdifferentiation of ATII cells. To assess whether this effect requires cell to cell contact, or it is mediated by secreted angiocrine factors, we collected the conditioned medium of alveolar lung ECs purified from either wt or *Flt1 KO*^*iEC*^ mice. In parallel, we purified ATII cells from both controls and bleomycin-treated mice and exposed them to the collected EC supernatants. We observed that control ATII cells invariably transdifferentiated into ATI cells, as expected, but the efficiency of their transdifferentiation increased by 40% and 200% when treated with supernatants from wt ECs and *Flt1* KO ECs, respectively (Fig. 4A, B). As described above, ATII cells purified from bleomycin-treated mice were not prone to transdifferentiation. The exposure to wt EC medium significantly increased the number of Rage^+^ ATI cells (Fig. 4C, D). However, they retained an irregular, not fully flattened morphology, indicative of incomplete differentiation. When bleomycin-exposed ATII cells were treated with medium collected from *Flt1* KO ECs, full transdifferentiation was observed, resulting in a quantity and morphology of ATI cells comparable to those derived from healthy mice (Fig. 4C, D). These results confirmed a paracrine effect of ECs on transdifferentiation of ATII cells, with an additional advantage gleaned from the knockout of the miR-200c-3p target *Flt1*.

To further confirm a paracrine crosstalk between alveolar ATII and ECs, we set-up a novel ex vivo cellular assay, which mimics the physiological interaction of these two cell types in the lung, where they are separated by a basal membrane. In this assay, ECs were first seeded on the lower side of a transwell membrane. Next, the transwell was turned upside down and ATII cells were seeded on the upper side of the same membrane. Using this system, we cultured ECs from wt and *Flt1 KO*^*iEC*^ mice together with healthy ATII cells. In all conditions, ATII cells efficiently transdifferentiated into Rage^+^ ATI cells, with a slight increase in the number of fully differentiated cells in the presence of *Flt1* KO ECs (Fig. 4E, G). When we repeated the experiment with ATII cells from bleomycin-treated mice, we found that these cells were unable to fully differentiate, either alone or in proximity with wt ECs. However, *Flt1* KO ECs rescued ATII differentiation capacity (Fig. 4F, H). Thus, we added additional evidence in support of our hypothesis that angiocrine factors secreted by *Flt1* KO ECs can rescue transdifferentiation of stressed fibrotic ATII epithelial cells.

### Endothelial *Flt1* depletion prevents bleomycin-induced fibrosis in vivo

**Fig. 5:**
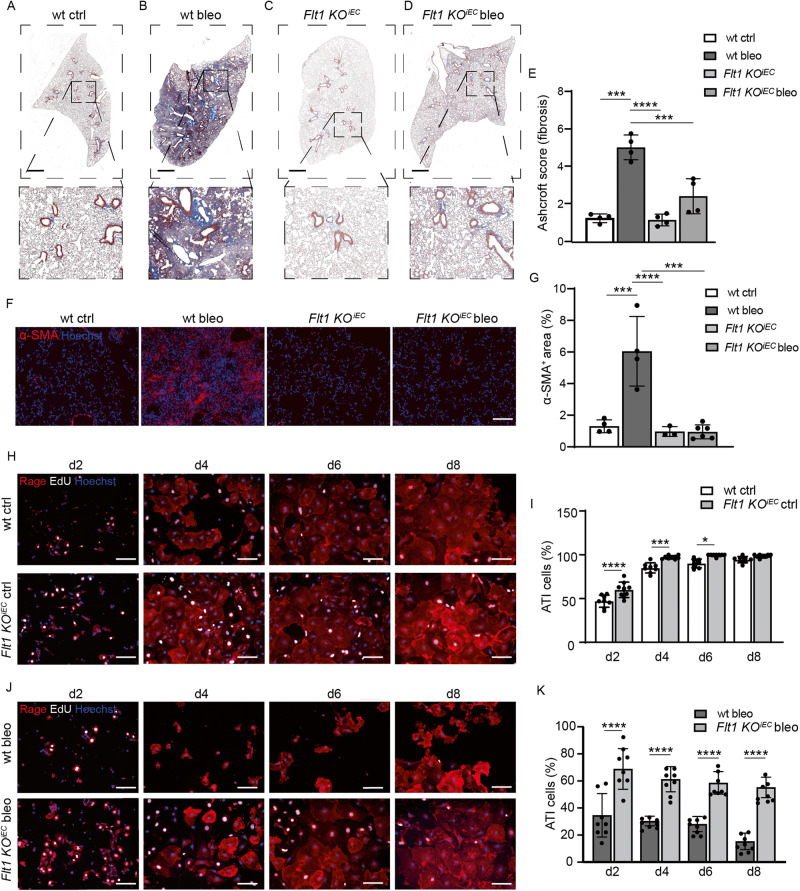
Endothelial *Flt1* depletion prevents bleomycin-induced fibrosis in vivo. Representative images of a control lung section stained with Masson-Trichrome to visualize collagen fibers in blue at 30 days after the administration of saline. High magnification is shown in the lower panel. **A** Representative images of a bleomycin-treated lung section stained with Masson-Trichrome to visualize collagen fibers in blue at 30 days after the administration of bleomycin. High magnification is shown in the lower panel. **B** Representative images of a lung section from *Flt1 KO*^*iEC*^ mice stained with Masson-Trichrome to visualize collagen fibers in blue at 30 days after the administration of saline. High magnification is shown in the lower panel. **C** Representative images of a lung section from *Flt1 KO*^*iEC*^ mice stained with Masson-Trichrome to visualize collagen fibers in blue at 30 days after the administration of bleomycin. High magnification is shown in the lower panel. **D** Quantification of pulmonary fibrosis using the Ashcroft score; *n* = 4. **E** Representative immunofluorescence images of lung sections stained for α-SMA. Nuclei were counterstained with Hoechst (blue). **F** Quantification of the area covered by α-SMA^+^ activated fibroblasts; *n* ≥ 3. **G** Representative immunofluorescence images of primary murine ATII cells harvested from wt or *Flt1 KO*^*iEC*^ mice and kept in culture for the indicated time points. ATII cells were stained for Rage (red) and for EdU (white). Nuclei were counterstained with Hoechst (blue). **H** Quantification of the transdifferentiation of ATII into ATI cells harvested from wt or *Flt1 KO*^*iEC*^ mice, shown as the percentage of Rage^+^ cells at the indicated time points; *n* = 8. **I** Representative immunofluorescence images of primary murine ATII cells harvested from wt or *Flt1 KO*^*iEC*^ mice after 30 days bleomycin administration and kept in culture for the indicated time points. ATII cells were stained for Rage (red) and for EdU (white). Nuclei were counterstained with Hoechst (blue). **J** Quantification of the transdifferentiation of bleo ATII into ATI cells harvested from wt or *Flt1 KO*^*iEC*^ mice, shown as the percentage of Rage^+^ cells at the indicated time points; *n* = 8. Scale bar in **A**,–**D** 1 mm for whole lung section and 100 μm for high magnification images; in **F**, **H**, **J**, 100 μm. Data in **E**, **G**, **I**, and **K** are shown as mean ± S.E.M. Statistical significance was determined using one- or two-way ANOVA, *P < 0.05, ***P < 0.001, ****P < 0.0001.

We then evaluated whether endothelial *Flt1* had an impact on pulmonary fibrosis development in vivo. For this, *Flt1 KO*^*iEC*^ and control mice were treated intratracheally with either bleomycin or saline, as a control. As expected, Masson trichrome staining demonstrated normal alveolar structure in both control and *Flt1 KO*^*iEC*^ mice (Fig. 5A, C), indicating that in vivo *Flt1 KO* in ECs per se does not affect lung structure. While lung parenchyma was almost completely replaced by fibrotic tissue at day 30 after bleomycin administration in wt mice (Fig. 5B), *Flt1 KO*^*iEC*^ mice developed minimal fibrosis with preserved lung structure (Fig. 5D), as documented by a 2.5-fold decrease in the Ashcroft score (Fig. 5E). Consistent results were obtained by staining activated fibroblasts for α-SMA and quantifying their relative abundance in the various groups (Fig. 5F, G).

Based on our previous evidence, we hypothesized that this protective effect in vivo was mediated by increased epithelial transdifferentiation capacity. Thus, we harvested ATII cells from both wt and *Flt1 KO*^*iEC*^ mice at 30 days after either bleomycin or saline administration and assessed their differentiation potential over 8 days in culture.

By staining for the ATI marker Rage, we observed a progressive increase in the number of positive cells harvested from wt mice, indicative of efficient transdifferentiation of ATI cells in culture (Fig. 5H, I). This process was even more pronounced in cells harvested from *Flt1 KO*^*iEC*^ mice, consistent with their increased ability to give rise to fully differentiated ATI cells.

After bleomycin administration, ATII cells from wt mice were not able to efficiently differentiate, as expected and according to the data shown in Fig. 1. However, the same cells from *Flt1 KO*^*iEC*^ mice preserved their capacity to differentiate into ATI cells even upon bleomycin treatment, reaching a number of Rage^+^ cells at day 8 comparable to cells harvested from saline-injected mice (Fig. 5J, K). Similar levels of transdifferentiation were obtained by assessing the expression of ATI cell markers (Supplementary Fig. 6).

To investigate whether EC-specific *Flt1* KO also resulted in increased epithelial cell proliferation, we added the nucleotide analog ethynyl deoxyuridine (EdU) to label the nucleus of dividing cells. We found that ATII cells from *Flt1 KO*^*iEC*^ mice proliferated more than those from wt mice both in control conditions and upon bleomycin administration, resulting in a significant increase in the percentage of Rage^+^EdU^+^ cells at day 8 (Supplementary Fig. 7A, B).

Thus, EC-specific deletion of *Flt1* results in an initial wave of proliferation and subsequent transdifferentiation of epithelial cells in both physiological and pathological conditions.

### Proteins secreted by Flt1 KO endothelial cells modulate epithelial transdifferentiation

**Fig. 6:**
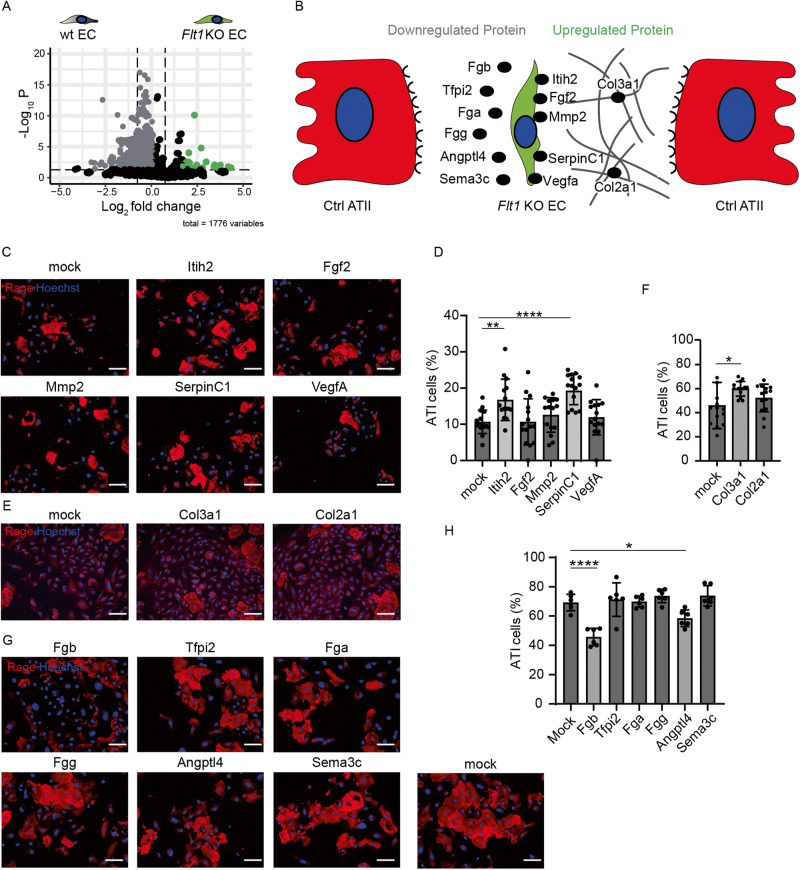
Proteins secreted by *Flt1* KO endothelial cells modulate epithelial transdifferentiation. **A** Volcano plot displaying differential expressed proteins between wt and *Flt1* KO ECs. The vertical axis (y-axis) corresponds to the mean expression value of log 10 (*q*-value), and the horizontal axis (x-axis) displays the log 2-fold change value. The gray and the green dots represent the proteins whose expression is differential expressed. Positive x-values represent up-regulation (log fold change > 0.75) and negative *x*-values represent down-regulation (log fold change < −0.75). Black dots represent proteins whose expression is not differential expressed. **B** Schematic representation of the interactome between ATII and *Flt1* KO ECs. **C** Representative immunofluorescence images of primary murine ATII cells harvested at 30 days after bleomycin administration and treated with the indicated conditions. ATII cells were stained for Rage (red). Nuclei were counterstained with Hoechst (blue). **D** Quantification of fully differentiated (>1500 µm^2^) Rage^+^ cells ATI cells, expressed as a percentage of Rage ^+^ cells, in the indicated conditions. **E** Representative immunofluorescence images of primary murine ATII cells harvested at 30 days after bleomycin administration and seeded with indicated matrix. ATII cells were stained for Rage (red). Nuclei were counterstained with Hoechst (blue). **F** Quantification of fully differentiated (>1500 µm^2^) Rage^+^ cells ATI cells, expressed as a percentage of Rage ^+^ cells, in the indicated conditions. **G** Representative immunofluorescence images of primary murine ATII cells treated with indicated conditions. ATII cells were stained for Rage (red). Nuclei were counterstained with Hoechst (blue). **H** Quantification of fully differentiated (>1500 µm^2^) Rage^+^ cells ATI cells, expressed as a percentage of Rage^+^ cells, in the indicated conditions. Scale bar in **C**, **E**, **G**, 100 μm. Data in **D**, **F**, and **H** are shown as mean ± S.E.M (*n* ≥ 3). Statistical significance was determined using one-way ANOVA, *P < 0.05, **P < 0.01, ****P < 0.0001.

To shed light on the molecular mechanism by which *Flt1* KO ECs promote the differentiation of ATII into ATI cells and thus prevents fibrosis, we interrogated the secretome of these cells by mass spectrometry analysis. In particular, we purified ECs from wt and *Flt1 KO*^*iEC*^ mice and cultured them in serum-free conditions for 3 days. Supernatants were collected, filtered, and purified proteins were analyzed by mass spectrometry (LC-MS/MS). A total of 820 proteins were identified as differentially expressed between wt and *Flt1* KO ECs (Supplementary File 1). Among these, 773 were downregulated and 47 upregulated, as represented in the volcano plot in Fig. 6A. As we observed a positive paracrine effect of *Flt1* KO on epithelial differentiation potential, we initially focused on upregulated proteins. We built the interactome between lung endothelial and epithelial cells, by merging our results with an available single-cell RNA sequencing dataset [22]. In this way, we identified 7 proteins that were upregulated in *Flt1* KO ECs and express a matching receptor on ATII cells as shown schematically in Fig. 6B.

Next, we validated the functional effect of the identified proteins, which entailed five soluble factors (Itih2, Fgf2, Mmp2, SerpinC1, VegfA) and two matrix components (Col3a1, Col2a1). To obtain a medium enriched in each of the soluble factors, we transfected HEK293T cells with the corresponding expression plasmids, using GFP as a control. After 2 days, each supernatant was collected and added to freshly isolated ATII cells from bleomycin-treated mice in the presence of EdU. We found that both SerpinC1 and Itih2 significantly increased ATII cell proliferation (Supplementary Fig. 8) and transdifferentiation (Fig. 6C, D). In parallel, we evaluated the biological effect of the two matrix components upregulated in *Flt1* KO ECs, both of which are collagen-related proteins. We overexpressed these proteins in 3T3-NIH fibroblasts, culturing the cells for 10 days to allow for matrix secretion and deposition, and then decellularized the culture dishes. Both control and collagen-coated dishes were then seeded with ATII cells from bleomycin-treated mice. As shown in Fig. 6E, F Col3a1 slightly but significantly promoted the transdifferentiation of ATII cells.

As most of the proteins were downregulated in *Flt1* KO compared to wt ECs, we also assessed the effect of the 6 most down-regulated proteins (Fig. 6B) and found that Angiopoietin-like 4 (Angptl4) and Fibrinogen β chain (Fgb), which was top in the list, significantly inhibited ATII to ATI transdifferentiation (Fig. 6G, H).

In conclusion, the knockout of *Flt1* in ECs results in both increased secretion of proteins that stimulate epithelial transdifferentiation (SerpinC1, Itih2 andCol3a1) and reduced secretion of proteins that inhibit epithelial transdifferentiation (Angptl4 and Fgb).

### Silencing of *Flt1* in human lung ECs promotes the transdifferentiation of ATII cells from IPF patients

To prove the relevance of our findings for human pulmonary fibrosis, we assessed whether *Flt1* depletion in human lung microvascular ECs improved the transdifferentiation of epithelial cells harvested from both healthy individuals and IPF patients.

**Fig. 7:**
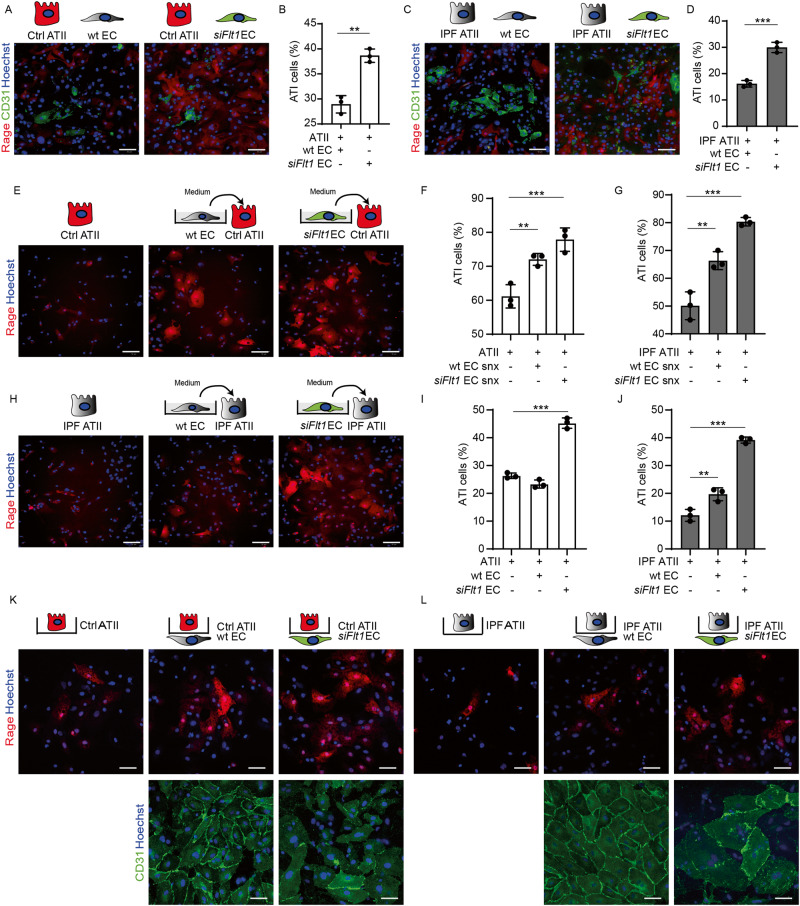
Flt1 depletion in human endothelial cells improves human diseased ATII cell transdifferentiation. **A** Representative immunofluorescence images of ATII cells from healthy donors kept in culture with human ECs transfected with either scramble (wt) or Flt1 siRNA, stained for CD31 (green) and Rage (red). Nuclei were counterstained with Hoechst (blue). **B** Quantification of fully differentiated (>1500 µm^2^) Rage^+^ ATI cells, expressed as a percentage of Rage^+^ cells, in the indicated conditions. **C** Representative immunofluorescence images of ATII cells from IPF patients, kept in culture with human ECs transfected with either scramble (wt) or Flt1 siRNA, stained for CD31 (green) and Rage (red). Nuclei were counterstained with Hoechst (blue). **D** Quantification of fully differentiated (>1500 µm^2^) Rage^+^ cells ATI cells from IPF patients, expressed as a percentage of Rage^+^ cells, in the indicated conditions. **E** Representative immunofluorescence images of healthy ATII cells conditioned with the supernatant of human ECs transfected with either scramble (wt) or Flt1 siRNA, stained for Rage (red). Nuclei were counterstained with Hoechst (blue). **F** Quantification of fully differentiated (>1500 µm^2^) Rage^+^ cells ATI cells from healthy donors, expressed as a percentage of Rage^+^ cells, in the indicated conditions. **G** Quantification of fully differentiated (>1500 µm^2^) Rage^+^ cells ATI cells from IPF patients, expressed as a percentage of Rage^+^ cells, in the indicated conditions. **H** Representative immunofluorescence images of ATII cells from IPF patients, conditioned with the supernatant of human ECs transfected with either scramble (wt) or Flt1 siRNA, stained for Rage (red). Nuclei were counterstained with Hoechst (blue). **I** Quantification of fully differentiated (>1500 µm^2^) Rage^+^ ATI cells from healthy donors, expressed as a percentage of Rage^+^ cells, in the indicated conditions. **J** Quantification of fully differentiated (>1500 µm^2^) Rage^+^ ATI cells from IPF patients, expressed as a percentage of Rage^+^ cells, in the indicated conditions. **K** Representative immunofluorescence images of healthy ATII cells in contact with wt or *Flt1*-silenced ECs cells, stained for Rage (red) and CD31 (green), respectively. Nuclei were counterstained with Hoechst (blue). **L** Representative immunofluorescence images of IPF ATII cells in contact with wt or *Flt1*-silenced ECs cells, stained for Rage (red) and CD31 (green), respectively. Nuclei were counterstained with Hoechst (blue). Scale bar in **A**, **C**, **E**, **H**, **K**, **L** 100 μm. Data in **B**, **D**, **F**, **G**, **I**, and **J** are shown as mean ± S.E.M (*n* ≥ 3). Statistical significance was determined using one-way ANOVA, **P < 0.01, ***P < 0.001.

First, we cultured healthy ATII cells together with lung ECs transfected with either a scramble or a *Flt1*-specific siRNA (Fig. 7A), which resulted in >90% downregulation of *Flt1* mRNA by RT-PCR, and found that *Flt1* silencing in ECs promoted the expression of Rage by ATII cells, indicating their increased transdifferentiation into ATI cells (Fig. 7B). Then, we repeated the experiment using ATII cells from IPF patients and again observed increased transdifferentiation in co-culture with Flt1-silenced ECs (Fig. 7C, D).

Second, we confirmed that this effect was mediated by the paracrine activity of secreted factors and transferred the medium collected from either control or Flt1-silenced ECs on top of ATII cells (Fig. 7E). Also in this case, we observed a pro-differentiation activity of EC media on both healthy and IPF ATII cells, which was further enhanced by *Flt1* silencing (shown for health ATII cells in Fig. 7E, F and for IPF ATII cells in Fig. 7G, H).

Finally, we used our transwell model to mimic the spatial relation of epithelial and endothelial cells on either side of the basal membrane in vivo. Consistent with our previous findings, Flt1 silencing in ECs promoted the transdifferentiation of both healthy and IPF ATII cells (Fig. 7I–L). Control ECs had a pro-differentiation effect on IPF, but not on healthy ATII cells, further pointing to Flt1 as a specific therapeutic target in the therapy of human pulmonary fibrosis.

## Discussion

In this work we show for the first time that lung ECs control epithelial regeneration in a paracrine manner and that this crosstalk can be exploited therapeutically to promote lung repair and inhibit fibrosis after damage. In particular, we show that the in vivo delivery of miR-200c-3p prevents but also reverts pulmonary fibrosis and that this goes beyond the known effect of miR-200c-3p on either fibroblast or epithelial cells [15]. We identify ECs as the major cellular target of miR-200c, which down-regulates *Flt1* and thus induces the secretion of both soluble and matrix proteins, i.e. SerpinC1, Itih2 and Col3a1, which in turn promote ATII cell activation and transdifferentiation into ATI cells.

Consistent with our previous data and additional publications showing that human ATII cells transdifferentiation is impaired in IPF patients [14, 23], here we confirmed that a similar phenotype is induced in mice by the administration of bleomycin, which represents the most widely accepted model of pulmonary fibrosis [24].

As we previously demonstrated that miR-200c-3p rescues this transdifferentiation defect in human IPF ATII cells, we wished to explore whether this also happens in mice upon bleomycin treatment. So far, miR-200 family members have been characterized for their anti-fibrotic properties, largely ascribed to reduced fibroblast activation and inhibition of EMT, a major driver of fibrosis [15, 25]. Both these mechanisms could justify the capacity of these miRNAs to prevent the onset of fibrosis when administered together with bleomycin. Our data instead indicate that miR-200c-3p is also able to act therapeutically and promote lung repair once fibrosis is established. This suggests that additional mechanisms of action beyond EMT might be responsible for its therapeutic effect. Specifically, we observed that miR-200c-3p promotes the transdifferentiation of ATII cells, unlike other members of the same family, such as miR-200a and miR-141, which block EMT but do not act on ATII transdifferentiation capacity [14].

To shed light on the molecular mechanism potentially responsible for this therapeutic activity, we interrogated miRTarBase and searched for validated targets of miR-200c. Besides *Zeb1* and *Zeb2*, known mediators of EMT, we found *Flt1* [19], previously reported to be upregulated in the broncho-alveolar lavage fluid from IPF patients [21]. Flt1, also known as VEGF receptor 1, is a tyrosine kinase receptor, which is expressed almost exclusively on vascular ECs [21]. Thus, we wondered whether miR-200c-3p could target ECs to down-regulate *Flt1* and thereby promote ATII cell transdifferentiation in a paracrine manner. This idea is consistent with recent studies describing a role for miR-200c in cancer-associated angiogenesis [26–28], and with other studies highlighting the role of angiogenesis during alveolar regeneration [8, 9, 29].

Our data are in line with a recent study showing that *Flt1* signaling drives the up-regulation of SDF-1 and CXCR4 in the lung, resulting in aberrant angiogenesis and fibrosis [30]. These data were obtained in transgenic mice globally expressing a *Flt1* variant that lacks the tyrosine kinase domain, and did not show which cell type expresses *Flt1* to trigger inflammation and fibrosis upon bleomycin administration. By using an EC specific knockout of *Flt1*, our study demonstrated that the absence of Flt1 specifically in ECs reduces the formation of fibrosis in vivo. We further observed that ATII cells harvested from bleomycin-treated *Flt1 KO*^*iEC*^ mice preserve their ability to transdifferentiate into ATI cells, indicating that Flt1 in ECs hampers ATII transdifferentiation upon lung injury.

To validate the existence of angiocrine communication between lung ECs and ATII cells, we set up a series of ex vivo models. First, we delivered miR-200c-3p to lung ECs in culture and verified that it down-regulates *Flt1*. Then, we added ATII cells from either control or bleomycin-injected animals and found that miR-200c-3p-treated cells induce ATII cell transdifferentiation in both cases. Similar conclusions were driven by using additional assays, including the direct co-culture of wild-type and *Flt1* KO EC with healthy and bleomycin-treated ATII cells, and the transfer of EC-conditioned medium to ATII cells. We also set up a novel system in which ECs and ATII cells are separated by a transwell membrane, mimicking the presence of the basal membrane in the alveolus. All results consistently confirmed that ECs from *Flt1 KO*^*iEC*^ mice promote the transdifferentiation of ATII cells from healthy mice and, most importantly, from bleomycin-treated mice. Consistent results were obtained by repeating the three assays using human cells, including both human microvascular lung ECs and human ATII cells harvested from healthy individuals and IPF patients. These results provide evidence that the mechanisms described in our work are relevant for human disease and set ECs as an innovative target for both the diagnosis and the therapy of human pulmonary fibrosis.

Finally, we provide evidence that *Flt1 KO*^*iEC*^ mice are protected from fibrosis development upon bleomycin administration and that ATII cells harvested from these mice preserve their transdifferentiation potential. Having proven that *Flt1* KO ECs communicate to ATII cells in a paracrine manner to promote their transdifferentiation, we sought to identify the molecules that mediate this crosstalk. Thus, we performed a comparative proteomic analysis of the secretome of both wild type and *Flt1* KO lung ECs and identified 1776 differentially expressed proteins. Out of these, we selected seven up-regulated and six down-regulated proteins that acted as ligands for receptors expressed by ATII cells. We individually screened the capacity of these 13 proteins to modulate ATII cell phenotype in co-culture and identified SerpinC1, Itih2 and Col3a1 as the most potent ones in inducing transdifferentiation, and Fgb as the most potent one in suppressing transdifferentiation. Of translational relevance, SerpinC1, also known as antithrombin III, was found to be down-regulated in the lung of bleomycin-treated mice [31], consistent with abnormal coagulation and up-regulation of its receptor Syndecan1 by ATII cells in IPF patients [32, 33], while Fgb resulted consistently increased in the sera of IPF patients [34–36]. Thus, we concluded that both soluble factors and matrix components mediate the positive effect of EC-specific *Flt1* KO on ATII cell transdifferentiation.

Collectively, our results show for the first time that endothelial-to-epithelial crosstalk significantly modulates lung repair by promoting the differentiation of ATII into ATI cells. This positions ECs as an innovative target to treat pulmonary fibrosis and, more in general, any condition that could benefit from enhanced epithelial regeneration.

## Supplementary information

Supplemental Figures and Methods

Reproducibility checklist

Supplementary File 1

Original Data File

## Data Availability

All original data will be made available upon request.
