# Supplementary material for: Flt1 produced by lung endothelial cells impairs ATII cell transdifferentiation and repair in pulmonary fibrosis
Source: Cell Death Dis. 2023 Jul 15;14(7):437. doi: 10.1038/s41419-023-05962-2 (PMC10349845; doi:10.1038/s41419-023-05962-2)
Supplement: Supplementary file 1 — Supplemental Figures and Methods [file 41419_2023_5962_MOESM1_ESM.pdf]

Flt1 produced by lung endothelial cells impairs ATII cell transdifferentiation and repair in lung fibrosis

Supplementary Figures

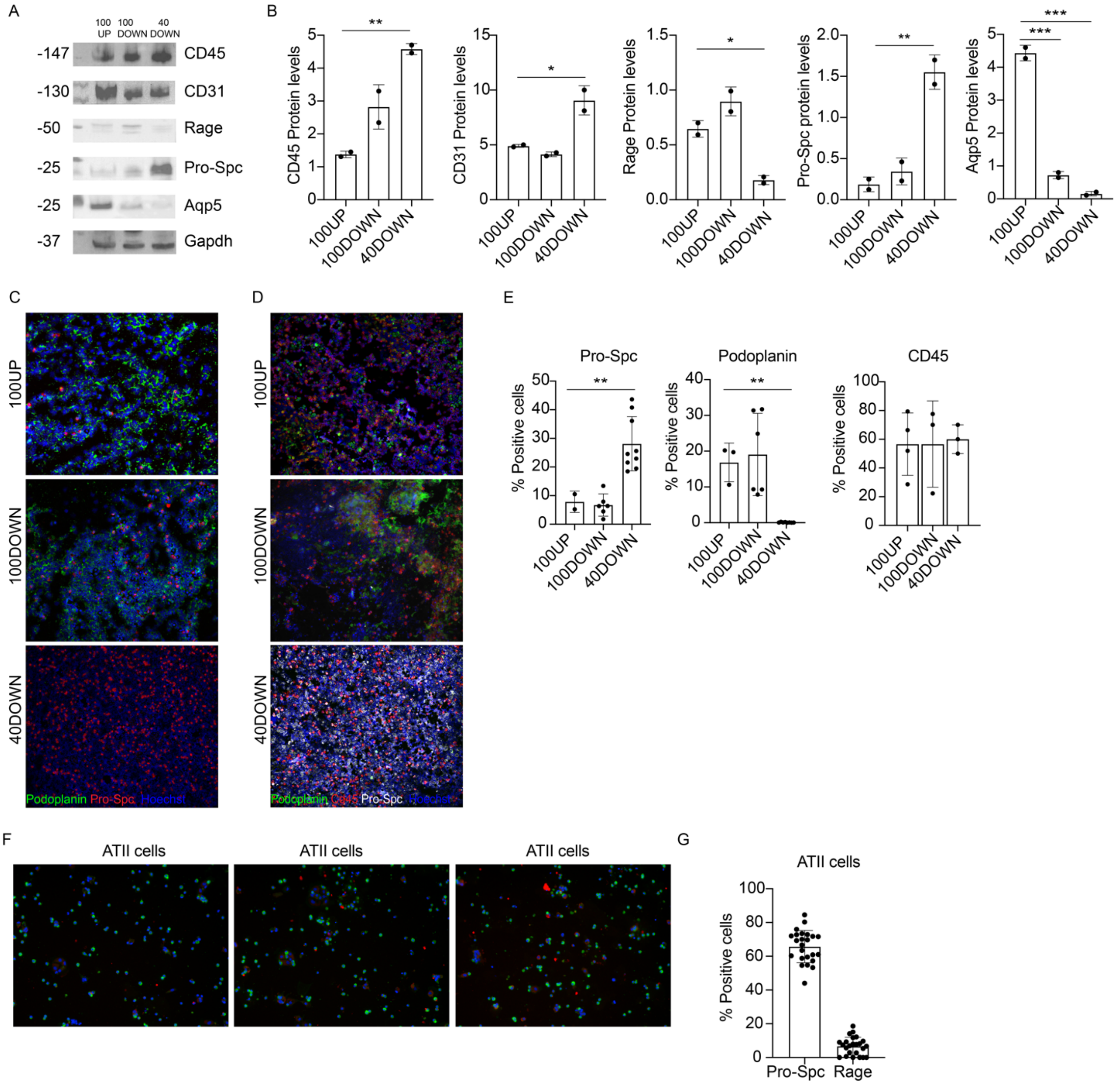

Supplementary Figure 1. Characterization of primary alveolar epithelial type II cells.

A. Representative Western Blots for the expression of CD45 (immune cell marker), CD31 (EC marker), Pro-Spc (ATII cell marker), Rage, Aqp5 (ATI cell markers) and Gapdh by cells that were retained in the upper (100UP) and lower (100DOWN) compartments of the 100 µm strainer, and in the final preparation obtained after additional filtering through a 40 µm strainer (40DOWN).

B. Quantification of Western Blot band intensity for the indicated proteins, normalized on Gapdh. Data are shown as mean ± S.E.M. Statistical significance was determined using one-way ANOVA, $*P < 0.05$, $**P < 0.01$, $***P < 0.001$.

C. Representative immunofluorescence images of cells attached to a microscope slide by cytospin centrifugation and stained for Podoplanin (green), Pro-Spc (red) to label ATI, ATII,

respectively. Nuclei were counterstained with Hoechst (blue).

D. Representative immunofluorescence images of cells attached to a microscope slide by cytospin centrifugation and stained for Podoplanin (green), Pro-Spc (white) and CD45 (red) to label ATI, ATII and inflammatory cells, respectively. Nuclei were counterstained with Hoechst (blue).

E. Quantification of percentage of Pro-Spc, Podoplanin and CD45 positive cells in the indicated strainer compartments. Data are shown as mean ± S.E.M. Statistical significance was determined using one-way ANOVA, $^{**}P < 0.01$.

F. Representative immunofluorescence images of cells stained for Pro-Spc (green) and Rage (red), to label ATII and ATI cells, respectively, in the final ATII cell preparation after 24 hours of culture.

G. Quantification of the percentage of Pro-Spc and Rage positive cells in the final ATII cell preparation after 24 hours of culture.

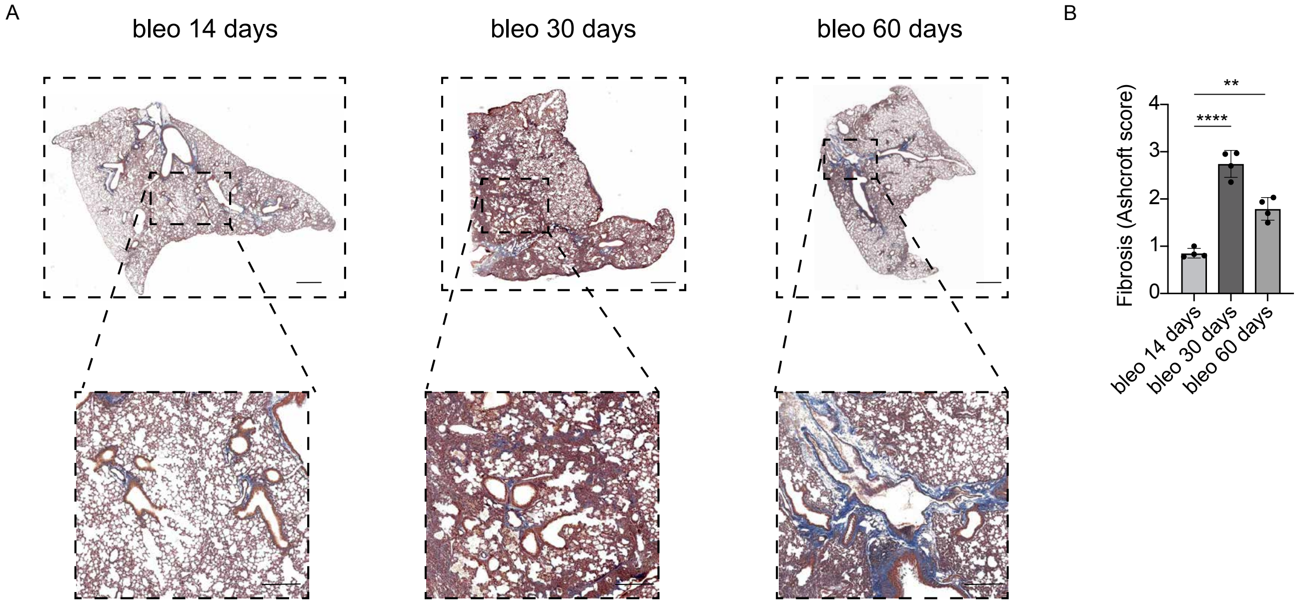

Supplementary Figure 2. Recovery of lung structure after bleomycin treatment.

A. Representative images of whole lung sections stained with Masson-Trichrome to visualize collagen fibers in blue at the indicated time points after the intratracheal administration of bleomycin. The inset shows a high magnification for each lung. Scale bar, 1mm in whole lung section and 100 μm in high magnification images.

B. Quantification of lung fibrosis using the Ashcroft score. Data are shown as mean ± S.E.M. Statistical significance was determined using two-way ANOVA, **P < 0. 01, ****P < 0. 0001.

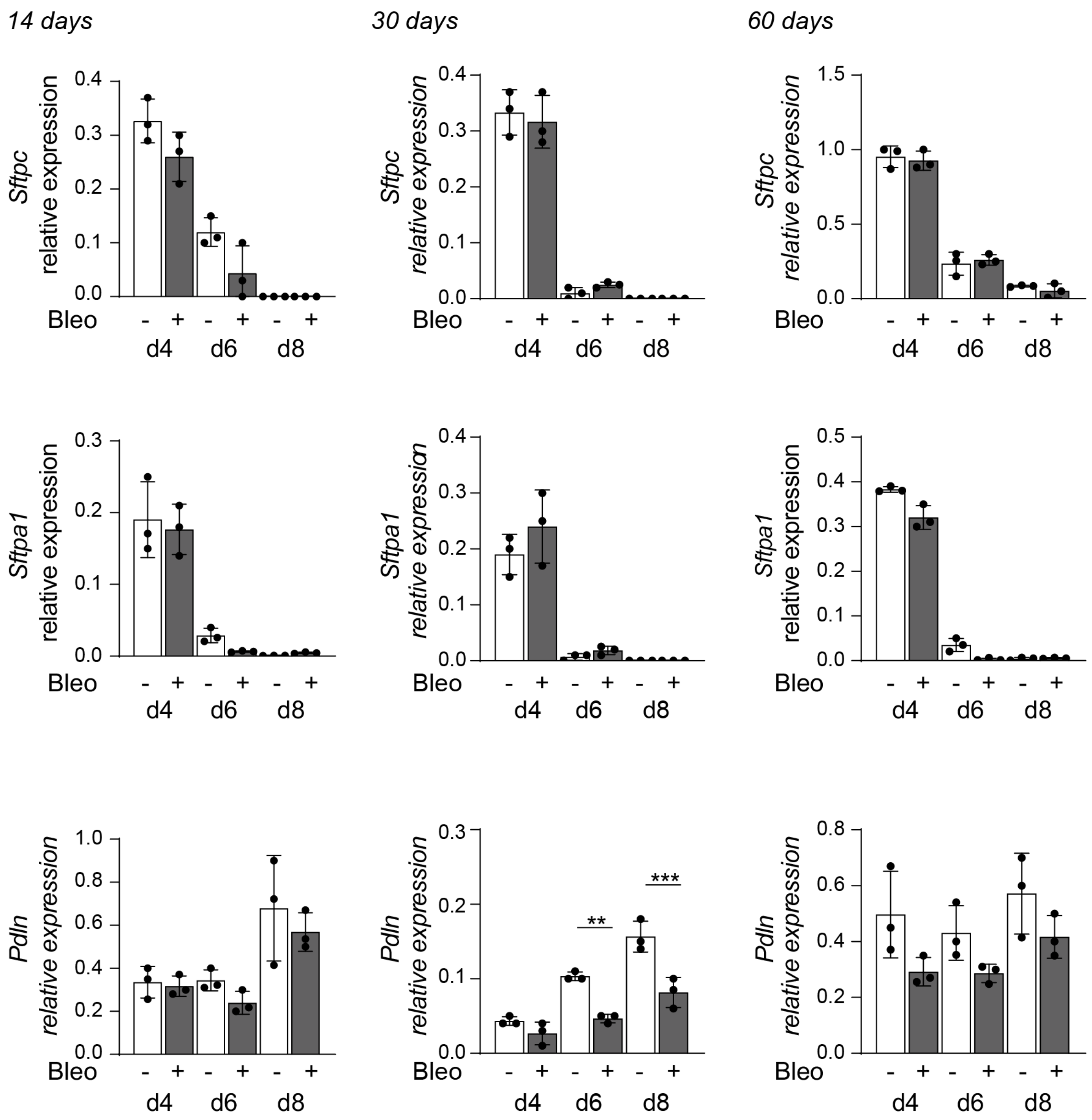

Supplementary Figure 3. ATII cells from bleomycin-treated mice show impaired transdifferentiation

Quantification of the expression levels of ATII (*Sftpc* and *Sftpa1*) and ATI (*Pdln*) cell markers by ATII cells harvested at 14, 30 and 60 days after PBS or bleomycin administration and kept in culture for the indicated time points.

Data are shown as mean ± S.E.M (n = 3). Statistical significance was determined using two-way ANOVA, $^{**}P < 0.01$, $^{***}P < 0.001$.

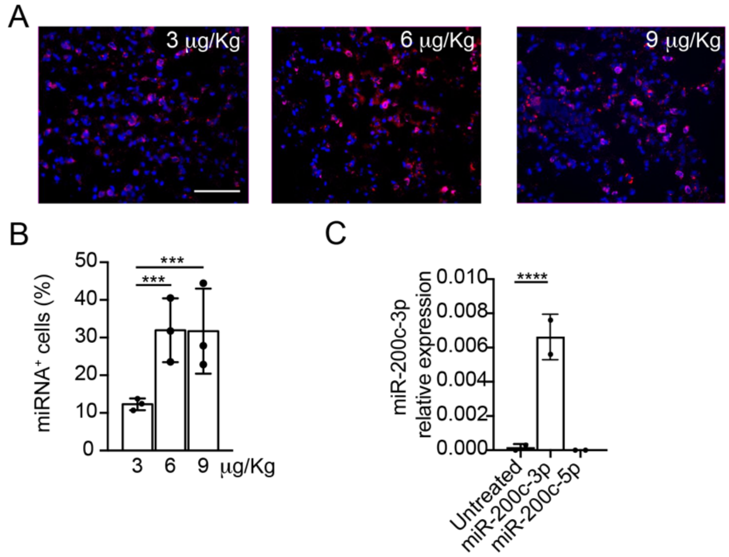

Supplementary Figure 4. Optimization of miRNA delivery to murine lungs

A. Representative immunofluorescent images of lung sections upon aerosol-mediated delivery of the indicated miRNA doses. miRNA is fluorescently labeled in red and nuclei are counterstained with Hoechst (blue). Scale bar, 100 mm.

B. Quantification of miRNA delivery to lung cells at the indicated miRNA doses. The intermediate dose (6 µg/Kg) was selected for further experiments.

C. Real-time PCR quantification of the expression levels of miR-200c-3p in lungs treated with miR-200c-3p mimic at 6 µg/Kg. Results are normalized to 5S.

Data in B, C are shown as mean ± S.E.M. Statistical significance was determined using a one-way ANOVA, ***P < 0.001, ****P < 0.0001.

A

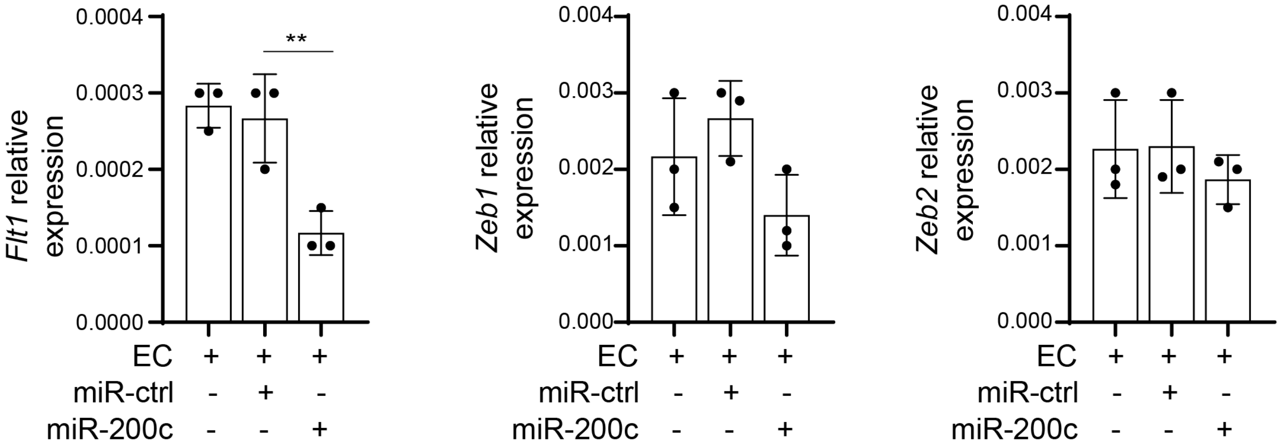

B

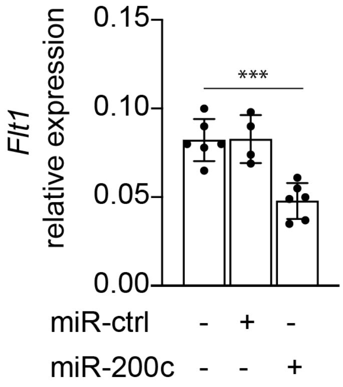

Supplementary Figure 5. *Flt1* is the most relevant target of miR-200c-3p.

A. Real-time PCR quantification of the expression levels of *Flt1, Zeb1* and *Zeb2* in primary alveolar ECs transfected with the indicated miRNAs.

B. Real-time PCR quantification of the expression levels of *Flt1* in lungs injected with indicated miRNAs.

Results are normalized to *Gapdh* expression. Data are shown as mean ± S.E.M. Statistical significance was determined using a one-way ANOVA, **P < 0.01, ***P<0.001.

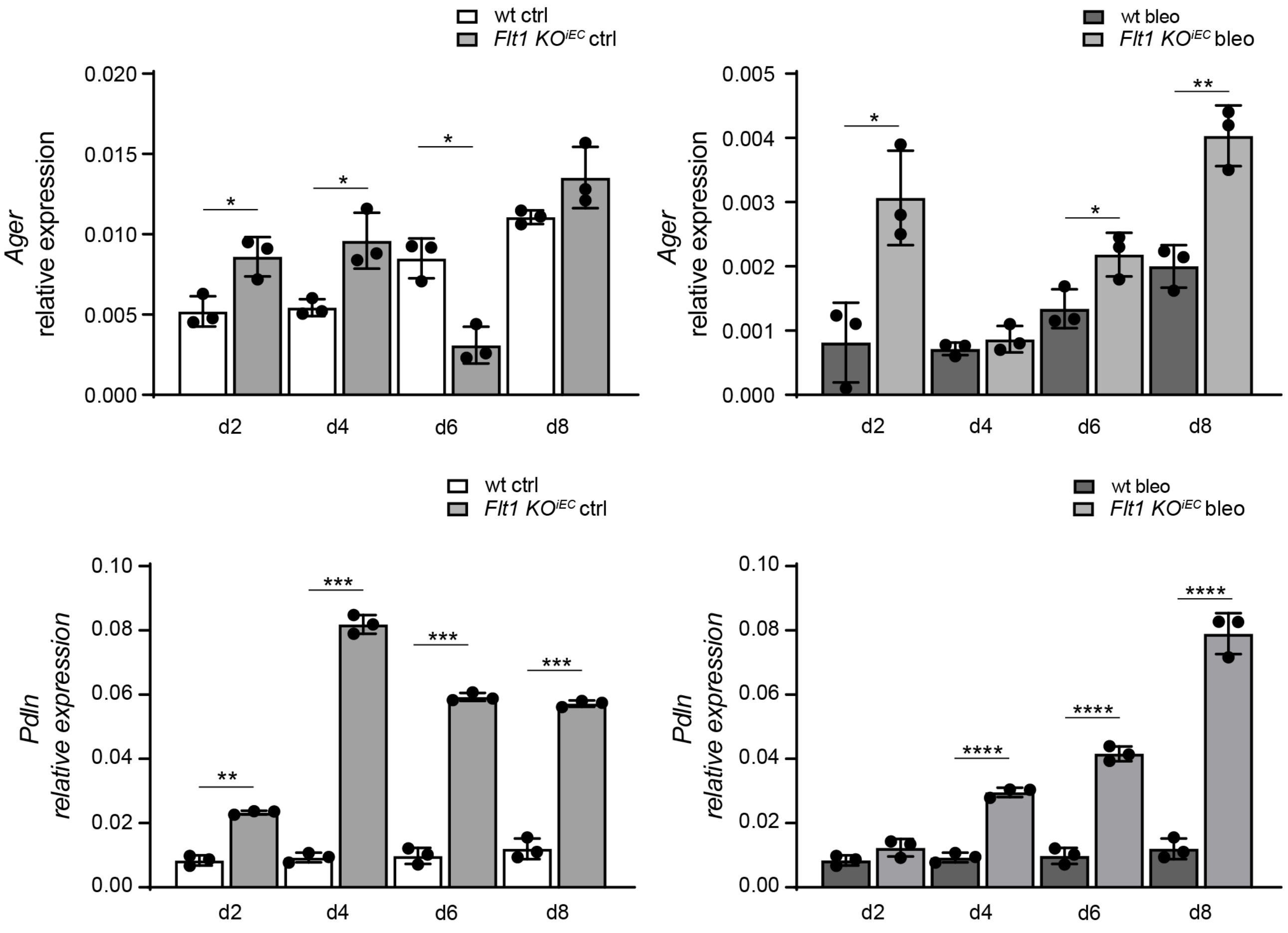

Supplementary Figure 6. Flt1 KO in ECs promotes the transdifferentiation of ATI cells from bleomycin-treated mice

Quantification of the expression levels of the ATI markers Ager and Podoplanin (Pdln) by ATII cells harvested from bleomycin-treated mice and kept in culture for the indicated time points.

Data are shown as mean ± S.E.M (n = 3). Statistical significance was determined using two-way ANOVA, *P < 0.05, **P < 0.01, ***P < 0.001.

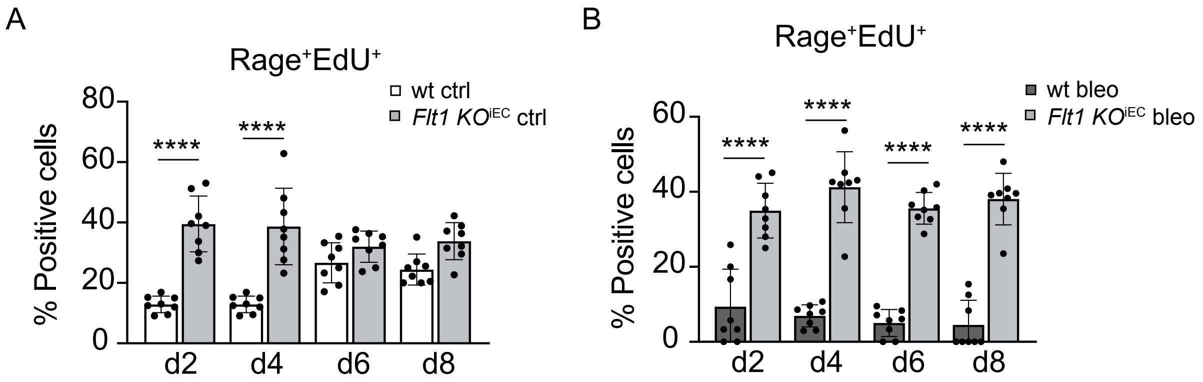

Supplementary Figure 7. Endothelial *Flt1* depletion promotes epithelial proliferation.

A. Quantification of proliferation and transdifferentiation of ATII into ATI cells harvested from either wt or *Flt1 KO^iEC^* mice, shown as the percentage of $Rage^{+}EdU^{+}$ at the indicated time points.

B. Quantification of the proliferation and transdifferentiation of ATII into ATI cells harvested from bleomycin-treated wt or *Flt1 KO^iEC^* mice, shown as the percentage of $Rage^{+}EdU^{+}$ at the indicated time points.

Data are shown as mean ± S.E.M. Statistical significance was determined using a two-way ANOVA, ****P < 0.0001.

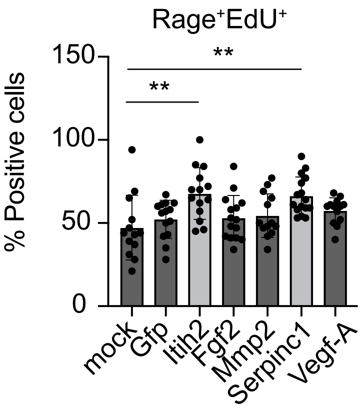

Supplementary Figure 8. SerpinC1 and Itih2 produced by *Flt1* KO endothelial cells promote epithelial proliferation.

Quantification of proliferation and transdifferentiation of ATII into ATI cells, expressed as a percentage of $Rage^{+}EdU^{+}$ cells, in the indicated conditions. Data are shown as mean ± S.E.M. Statistical significance was determined using a two-way ANOVA, $^{**}P < 0.01$.

Supplementary Methods

Primary cells

Murine ATII isolation. Primary murine ATII cells were isolated from distal lung of C57BL/6 mice by tissue digestion with dispase (BD, Biosciences) and mechanically dissociation using a McIlwain tissue chopper (Metrohm USA), as reported (15). Dissociated tissue was treated with 40 µg/ml DNAse I (Sigma-Aldrich) and passed first through a 100 µm cell strainer, then through a 40 µm cell strainer and eventually centrifuged at 450 x g. Mesenchymal cells were removed by pre-plating and inflammatory cells were removed by negative selection using simultaneous incubation with a Dynabeads Untouched Mouse T Cells Kit (11413D) and Dynabeads Mouse DC Enrichment Kit (11429D, Invitrogen). ATII cells were seeded on plates coated with a mixture of gelatin (G9391, Sigma-Aldrich) and fibronectin (33010-018, Invitrogen) in Pneuma-Cult medium (005001, Stem Cell) and stained with anti-Pro-Spc (ab90717, abcam) and anti-Rage (af1145, R&D) antibodies in 96 well-plates or on transwells. 5-ethynyl2'-deoxyuridine (EdU) was added to the medium (1:1000) to label proliferating cells and then visualized using Click-it EdU Cell Proliferation Kit (C10338, Thermo Fisher) following manufacturer instructions.

Murine EC isolation. Primary ECs were isolated from adult lungs of C57BL/6 mice. Lungs were removed, cut into small pieces and placed in CBFHH. The tissue was digested using Skeletal muscle dissociation kit (130-098-305, Miltenyi Biotec), for 30 minutes at 37°C. The solution was filtered using a 70 µm-pore size cell strainer and centrifuged at 400 x g. The pellet was resuspended in a solution containing PBS, 0,5% Bovine Serum Albumin (BSA) and 0,5 M EDTA and incubated with anti-CD45 magnetic MicroBeads (130-052-301, Miltenyi Biotec). CD45-negative cells were centrifuged at 400 x g for 10 minutes and incubated with anti-CD31 magnetic microbeads (130-097-418, Miltenyi Biotec). CD31-positive cells were washed and cultured in plates coated with a mixture of gelatin and fibronectin in EGM- 2 medium (CC- 3162, Lonza) in 96-well plates, together with ATII cells, or seeded on the lower side of a transwell and stained for Erg (ab92515, abcam).

Human cells. Human lung tissue from unidentified patients with IPF or recently deceased donors was obtained from the Lung Tissue Bank at Temple Lung Center (Dept of Thoracic Medicine and Surgery, Temple University, Philadelphia, PA, USA). ATII cells were isolated as we previously described, after obtaining written informed consent by patients and approval by the Institutional Review Board (approval 4407) at Partners Healthcare and Temple University (14). Human lung microvascular ECs were purchased from Lonza (CC-2527). For miRNA transfection, cells were plated in 96-well plates in EGM2 medium supplemented with 10% FBS without antibiotics. As a control for transfection efficiency a toxic siRNA targeting the polyubiquitin-C gene (M-019408-01, Dharmacon) was used. The day after seeding, a mixture of transfection reagent (Lipofectamine RNAiMAX, Thermo Fisher Scientific) and OPTI- MEM (Thermo Fisher Scientific) was incubated at room temperature for 5 minutes. Either si-Flt1 (M-003136-03-0005, Dharmacon) or a control, scrambled siRNA (D-001210-0X, Dharmacon) were added and the solution was incubated for 30 minutes at room temperature, and eventually added dropwise to the cells.

Transfection of miRNA in murine EC cells. Murine ECs were plated in 96-well plates. All miRNAs (miRIDIAN miRNA mimics) and siRNAs (siGENOME siRNA) were purchased from Dharmacon, Thermo Fisher Scientific. EC cell transfection was performed in EGM2 medium supplemented with 5% FBS without antibiotics and according to a standard transfection protocol with miR-200c-3p (HMI0354, Sigma) and a control miRNA (D-001210- 04-20, Dharmacon). As a control for transfection efficiency, a toxic siRNA targeting the polyubiquitin-C gene (M-019408-01, Dharmacon), was used together with a scrambled siRNA (D-001210-0X, Dharmacon). A mixture of transfection reagent (Lipofectamine RNAiMAX, Thermo Fisher Scientific) and OPTI-MEM (Thermo Fisher Scientific) was incubated at room temperature for 5 minutes. miRNAs at 25

μM final concentration were added, the solution was incubated for 30 minutes at room temperature, and eventually added dropwise to the cells.

Transfection of HEK293T and production of enriched supernatant. HEK293T cells were cultured at 70% confluence. A mixture of DNA (10 μg in a 60 mm dish), 60 μl of 2M $CaCl_2$ and water was added dropwise to 500 μl of 2X HEPES Buffered Saline (HBS) in agitation. After 10 minutes of incubation at RT, the transfection mix was added dropwise to the cells in antibiotic-free cell culture medium with 10% FBS. A plasmid encoding for EGFP was used as a positive control to check transfection efficiency (consistently higher than 90%). One day after transfection, the cell culture medium was replaced with PNEUMA-cult medium supplemented with 10% FBS and antibiotics and kept for one day. The enriched supernatant was recovered after 24 hours, centrifuged at 400 x g for 10 minutes to remove cell debris, filtered with a 0.22 μm cell strainer and stored at -80°C.

Transfection of 3T3-NIH fibroblasts and production of the extracellular matrix. 3T3-NIH cells were cultured at 70% confluence. Lipofectamine 3000 was diluted in OPTI-MEM medium. DNA (75 ng) was diluted in OPTI- MEM and P3000 reagent was added to the mixture. The diluted DNA was added to diluted Lipofectamine 3000 and the final mixture was incubated for 15 minutes at room temperature. After incubation, the transfection mix was added to 3T3-NIH cells. Transfection using a plasmid encoding for EGFP was used to check for transfection efficiency (consistently higher than 90%). Matrix production by fibroblasts was obtained as described by Franco- Barraza (16). Plates were pre-treated to stabilize anchoring of the matrix to the culture surface by pre-coating with 0.2% gelatin for 1 hour at RT. After washing with PBS, 1% glutaraldehyde (dissolved in PBS) was added for 30 minutes at RT and then washed with PBS. The plates were incubated with 1 M ethanolamine (dissolved in water) for 30 minutes at RT and washed carefully with PBS. Fibroblasts were cultured for 9 days in DMEM supplemented by 10% FBS, antibiotics, and 50 μg/ml of ascorbic acid. The day after cell seeding, fibroblasts were transfected with Col2a1 and Col3a1 plasmids. The medium was replaced every 48 hours. To decellularize the matrix, the culture medium was carefully removed and cells rinsed twice with PBS, then incubated with an extraction buffered composed by 0.5% Triton X-100, 20 mM NH_4OH in PBS for 10 minutes at 37°C. The extraction buffer was diluted with 100 μl of PBS and incubated overnight. The day after, the matrix was washed several times and ATII cells were seeded on top of it.

Animal experiments. All animal experiments were conducted in compliance with the European guidelines and international laws and policies, with the approval of ICGEB Animal Welfare Board, Ethical Committee, and Italian Ministry of Health (authorizations 538/2019 and 648/2019). Adult C57BL/6 and Cdh5-Cre; *Flt1 fl/fl* mice (male, 8 weeks old) were randomly assigned to groups of at least 9 animals, without blinding. Mice were housed under optimal environmental conditions in a temperature-controlled environment with 12/12 hours light/dark cycles. Bleomycin was injected intratracheally at 1 U/kg, miR-200c-3p was administered at 6 μg/kg. Mice were sacrificed 30 days after treatment. The severity of fibrosis was determined by histological analysis (17).

Histology and microscopy. Cells and tissue sections were fixed with 4% PFA, permeabilized by incubating with 0.1% Triton in PBS and then blocked with 5% BSA in PBS containing 0.1% Triton. Cells were incubated with primary (Pro-Spc, abcam #ab90716; Podoplanin, Hybridoma bank #ab531893; Erg, abcam #ab92513, Rage, R&D #af1145, CD45, abcam #ab10558, CD31, R&D #af3628, Aquaporin 5 (Aqp5), Millipore, #ab15858) and secondary antibodies (AlexaFluorTM anti goat IgG, Invitrogen, #A-11057, AlexaFluorTM anti rabbit IgG, Invitrogen, #A-21206, Goat Anti-Syrian Hamster IgG H&L (AlexaFluor 488), Invitrogen, Abcam #ab180063) and nuclei were stained using Hoechst 3334. Images were acquired using an Operetta high content screening microscope (Perkin Elmer) with a 20x objective, Nikon A1 Plus Microscope equipped

with DC-152Q-C00-FI using Nis 4.30 software (Nikon) with a 20x objective and with Zeiss LSM 880 Airyscan with a 20x objective. A minimum of 10 fields were acquired per well, and the number of Rage positive cells or Rage-EdU positive cells were quantified using the Image J and Columbus software. For tissue sections, immunohistochemistry was performed on paraffin embedded sections fixed with formalin, after deparaffinization before processing. For visualization of fibrosis, tissue was stained using Masson-Trichrome (Bio-Optica) and then acquired with D-sight slide scanner (Menarini).

qRT-PCR. RNA extraction was performed using Trizol (Invitrogen) and cDNA was prepared using the First strand cDNA synthesis kit (Thermo Scientific) following manufacturer instructions. Samples were run on a C1000 Thermal Cycler (Biorad). *Gapdh* was used as housekeeping gene. Primers were designed using the Blast software:

mFlt1: fw TTCATCAGTGTGAAACATGC, rev CGAGCCATCTTTTAACCATAC; mZEB1: fw GCTGGCAAGACAACGTGAAAG, rev GCCTCAGGATAAATGACGGC; mZEB2: fw ATTGCACATCAGACTTTGAGGAA, rev ATAATGGCCGTGTCGCTTCG;
mSftpc: fw ATGGACATGAGTAGCAAAGAGGT, revCACGATGAGAAGGCGTTTGAG
mSftpa1: fw CATCAGATTCTGCAAACAATGGG, rev GGCTCTGGTACACATCTCTCTAA
mAger: fw CTTGCTCTATGGGGAGCTGTA, rev CATCGACAATTCCAGTGGCTG
mPodoplanin: fw AGAGAACACGAGAGTACAACCA, rev CGTTTCATCCCCTGCATTATCT
mGapdh: fw TGACCTCAACTACATGGTCTACA, rev CTTCCCATTCTCGGCCTTG.
The small RNA fraction was isolated from mice tissue using the miRNeasy Mini Kit (Qiagen) according to the manufacturer instructions. DNase treatment was performed after RNA isolation. For miR-200c-3p and miR-200c-5p quantification, total RNA was reverse transcribed using miRCURY LNA PCR synthesis kit (Qiagen) and qRT-PCR was performed with pre-designed miRCURY LNA PCR primer sets (Qiagen) and miRCURY LNA SYBR Green mix according to the manufacturer's instructions. miRNA expression was normalized on the expression levels of 5S rRNA.

Western Blot. Protein lysates were run in 10% polyacrylamide gels and transferred to nitrocellulose membranes probed with the following antibodies: CD45, abcam #ab10558, CD31, R&D #af3628, Aquaporin 5 (Aqp5), Millipore, #ab15858, Rage, R&D #af1145, Pro-Spc, abcam #ab90716 and Gapdh, Thermo-Fisher Scientific #MA5-15738.

Mass Spectrometry. Conditioned medium (CM) was centrifuged at 2000 × g at 4 °C for 10 min and passed through a 0.2 µm filter to remove cellular debris. CM was then concentrated using a 3 kDa cut-off concentrator and precipitated with four volumes of pre-chilled acetone at – 20 °C overnight. Protein pellets were washed with 80% acetone, suspended in 6M urea and 100 mM ammonium bicarbonate pH 8, and protein concentration was quantified using bicinchoninic acid assay. Proteins (20 µg) were reduced using 10 mM dithiothreitol (DTT) for 45 min at room temperature followed by alkylation with 20 mM iodoacetamide (IAA) for 30 min in the dark. Proteins were then digested with 0.5 µg Lys-C (Promega, #VA1170) for 4 h at room temperature. Subsequently, the samples were diluted to 1:5 with 100 mM ammonium bicarbonate and digested with 1 µg of trypsin (Promega, #V5111) overnight. The day after, digestion was stopped with 1% trifluoroacetic acid. Samples were then desalted on in-house–packed StageTips (doi: 10.1038/nprot.2007.261), lyophilized in a Speed-vac and resuspended in 20 µL of 0.1% formic acid for LC-MS/MS analysis.

LCMS Method. Digested samples were separated on an Easy-nLC 1200 system (Thermo Fisher Scientific) by a 85 minutes gradients with a 400 nl/min flow rate on a 28 cm column with an inner diameter of 75 µm packed in-house with ReproSil-Pur C18-AQ material (3 µm particle size, Dr.

Maisch, GmbH), heated at 40°C. The gradient was set as follows: from 5% to 25% over 52 minutes, from 25% to 40% over 8 minutes and from 40% to 98% over 10 minutes at a flow rate of 400 nL/min. Buffers were 0.1% formic acid in water (A) and 0.1% formic acid in acetonitrile (B). Samples were analyzed in an Orbitrap Fusion Tribrid mass spectrometer (Thermo Fisher Scientific, San Jose, CA, USA) and data acquired in data-dependent mode (2100 V). Full scans were acquired in the Orbitrap performed at 120000 FWHM resolving power (at 200m/z) and an AGC target of 1x106, followed by a set of (higher-energy collision dissociation) MS/MS scans over 3 sec cycle time. A mass range of 350-1100 m/z was surveyed for precursors, with a maximum injection time of 50 ms and the first mass set at 140 m/z for fragments. The MS/MS scans were performed at a collision energy of 30%, 150 ms of maximum injection time (ion trap) and a target of 5x103 ions. Peptide searches were performed in Proteome Discoverer 2.2 software (Thermo Fisher Scientific) against the Mus musculus FASTA file (Uniprot, downloaded April 2021) and a database containing major common contaminants. Proteins were identified using MASCOT search engine with a mass tolerance of 10 ppm for precursors 0.6 Da for products. Trypsin was chosen as the enzyme with 5 missed cleavages, and static modification of carbamidomethyl (C) with variable modification of oxidation (M) and acetyl (protein N-term) were incorporated in the search. False discovery rate was filtered for <0.01 at PSM, peptide and protein level. A minimum number of two peptides per protein was set and results were filters to exclude potential contaminants.

Statistical analysis. GraphPad Prism software was used to calculate the statistical significance of the presented data. The reported results are expressed as mean value +/- standard error mean (SEM) and each experiment was performed 3 times with 3 biological replicates. One-way and two-way ANOVA statistics followed by Dunnet's multiple comparison test were used for multiple datasets. Significant values are indicated by the asterisks above the graphs (* P < 0.05, ** P < 0,01, *** P < 0,001, **** P < 0,0001).
