# Supplementary figures and images for: Flt1 produced by lung endothelial cells impairs ATII cell transdifferentiation and repair in pulmonary fibrosis
Source: Cell Death Dis. 2023 Jul 15;14(7):437. doi: 10.1038/s41419-023-05962-2 (PMC10349845; doi:10.1038/s41419-023-05962-2)

CD45

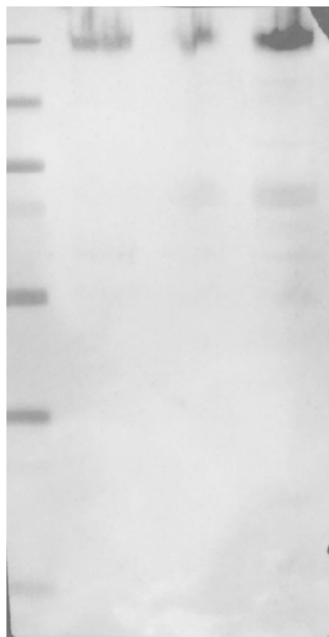

CD31

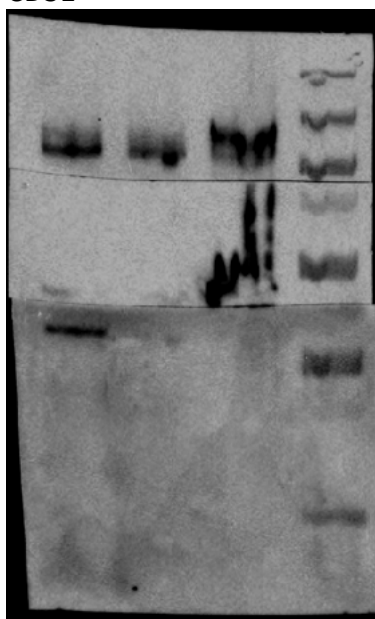

RAGE

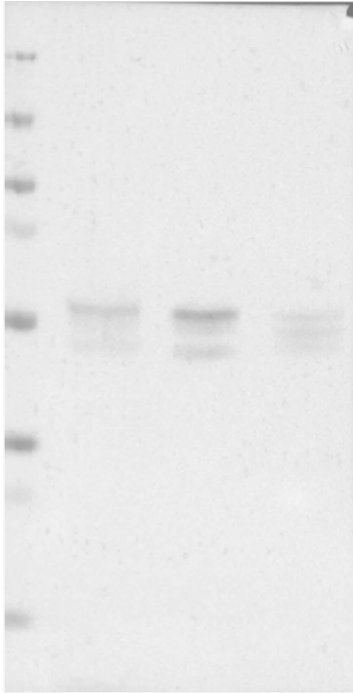

Pro-SPC

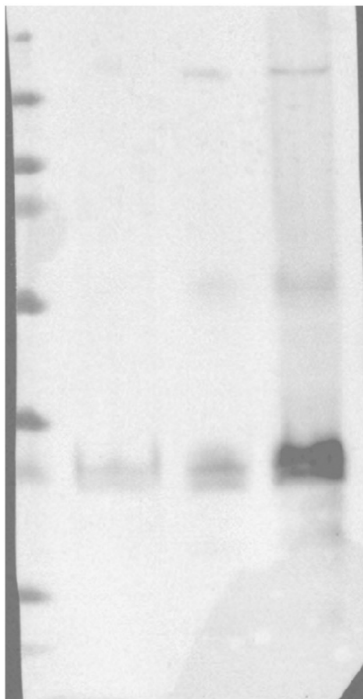

AQP5

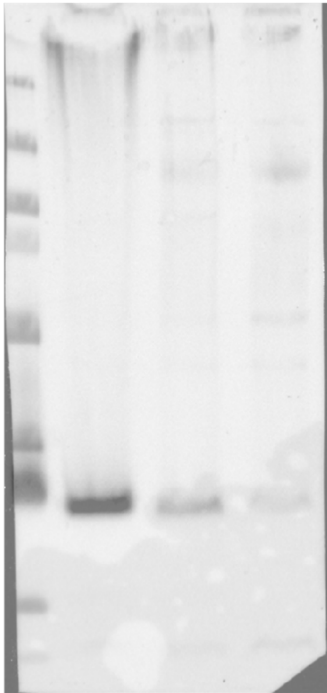

GAPDH

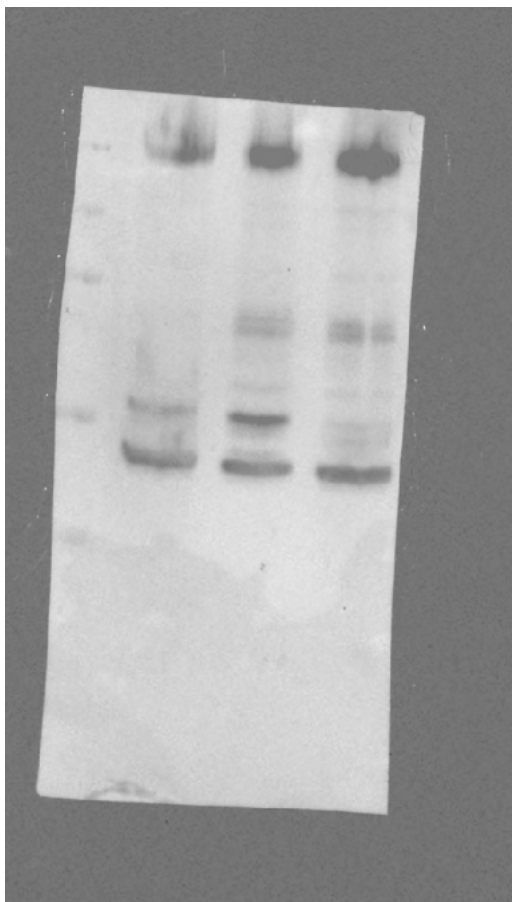

Supplement: Supplementary file 4 — Original Data File [file 41419_2023_5962_MOESM4_ESM.pdf]
